# Surgical and Trauma Capacity Assessment in Rural Haryana, India

**DOI:** 10.5334/aogh.3173

**Published:** 2021-02-12

**Authors:** Manisha B. Bhatia, Srivarshini C. Mohan, Kevin J. Blair, Marissa A. Boeck, Ashish Bhalla, Sristi Sharma, Irene Helenowski, Leah C. Tatebe, Benedict C. Nwomeh, Mamta Swaroop

**Affiliations:** 1 Indiana University, School of Medicine, Department of Surgery, Indianapolis, IN, US; 2 Cedars-Sinai Hospital, Department of Surgery, Los Angeles, CA, USA; 3 University of California Los Angeles, Department of Surgery, Los Angeles, USA; 4 University of California San Francisco, Department of Surgery, San Francisco, CA, USA; 5 Postgraduate Institute of Medical Education and Research, Chandigarh, IN; 6 University of Colorado, Department of Surgery, Denver, Colorado, USA; 7 Northwestern University Feinberg School of Medicine, Department of Preventative Medicine, Chicago, USA; 8 Northwestern University Feinberg School of Medicine, Department of Surgery, Chicago, USA; 9 Department of Trauma, Cook County Health, Chicago, IL, USA; 10 Surgeons Overseas, New York, NY, US; 11 Ohio State University, Nationwide Children’s Hospital, Department of Pediatric Surgery, Columbus, USA

## Abstract

**Background:**

Trauma is a major global health problem and majority of the deaths occur in low- and middle-income countries (LMICs), at even higher rates in the rural areas. The three-delay model assesses three different delays in accessing healthcare and can be applied to improve surgical and trauma healthcare delivery. Prior to implementing change, the capacities of the rural India healthcare system need to be identified.

**Objective:**

The object of this study was to estimate surgical and trauma care capacities of government health facilities in rural Nanakpur, Haryana, India using the Personnel, Infrastructure, Procedures, Equipment and Supplies (PIPES) and International Assessment of Capacity for Trauma (INTACT) tools.

**Methods:**

The PIPES and INTACT tools were administered at eight government health facilities serving the population of Nanakpur in June 2015. Data analysis was performed per tool subsection, and an overall score was calculated. Higher PIPES or INTACT indices correspond to greater surgical or trauma care capacity, respectively.

**Findings:**

Surgical and trauma care capacities increased with higher levels of care. The median PIPES score was significantly higher for tertiary facilities than primary and secondary facilities [13.8 (IQR 9.5, 18.2) vs. 4.7 (IQR 3.9, 6.2), p = 0.03]. The lower-level facilities were mainly lacking in personnel and procedures.

**Conclusions:**

Surgical and trauma care capacities at healthcare facilities in Haryana, India demonstrate a shortage of surgical resources at lower-level centers. Specifically, the Primary Health Centers were not operating at full capacity. These results can inform resource allocation, including increasing education, across different facility levels in rural India.

## Introduction

A disproportionally large number of the estimated five to six million annual deaths due to injury occur in low- and middle-income countries (LMICs). These deaths can be attributed partly to a lack of access to surgical care and a scarcity of emergency response systems and trauma services [1,2,3,4,5,6,7]. Improvement of surgical and trauma care in LMICs can reduce mortality due to injury [7]. Strengthening these systems with the three-delay interdisciplinary model, initially applied to reduce maternal mortality, can ultimately advance healthcare systems, thereby increasing overall access to healthcare [7]. This interdisciplinary model assesses three different delays in accessing healthcare: one in seeking care, one in reaching care, and one in receiving care [7]. The same model can guide improvements in LMIC surgical and trauma care systems with initiatives determined by existing capacities and deficits as determined by baseline evaluations [8].

India is a LMIC with a diverse population of nearly 1.3 billion people who speak over 22 languages. The median age is 27.6 years and the net population growth rate is 12 people per 1,000 people [9,10]. There is an average of 0.8 physicians per 1,000 people which pales in comparison to 3 physicians per 1,000 people in LMICs in Europe and Central Asia [11]. Due to the paucity of trained physicians, India’s National Rural Health Mission (NRHM) developed a tiered system ranging from basic subcenters managed by nurses to tertiary health centers staffed with physicians of every specialty. The NRHM has tried to expand the reach of healthcare by employing community health workers (ASHAs) to provide antenatal and postnatal care, family planning, sanitation and hygiene education, iron and folic acid supplements, and referrals of malnourished patients to nearby Primary Health Centers.

The ASHAs have provided a strong link between community members and primary care but there remains a disconnect between community members and surgical and trauma healthcare. Even with the several surgical and trauma care capacity evaluations conducted throughout South Asia, there is no baseline surgical capacity data in India [12,13,14,15,16]. This study aims to quantitatively assess surgical and trauma care capacities of government health facilities serving the community of Nanakpur, Haryana, India using the Personnel, Infrastructure, Procedures, Equipment and Supplies (PIPES) and International Assessment of Capacity for Trauma (INTACT) tools. Results will guide initiatives to strengthen surgical systems in Nanakpur by addressing the delays highlighted in the interdisciplinary model.

## Methods

### Setting

Haryana is one of 29 states in India and is in the northern half of the country. Sixty-five percent of Haryana’s population lives in rural areas [17], similar to 67% of the entire Indian population [18]. Nanakpur is a rural community of 37,168 people within the state of Haryana, located approximately 30 km northeast of Haryana’s capital, Chandigarh [19]. Two of the authors previously completed a general needs assessment in Nanakpur in 2012 that revealed the community’s unique composition of both farmers and brick manufacturing laborers. The study further concluded that the community faces both economic and geographic barriers in accessing healthcare [20].

### Health Facility Selection

**Figure 1:**
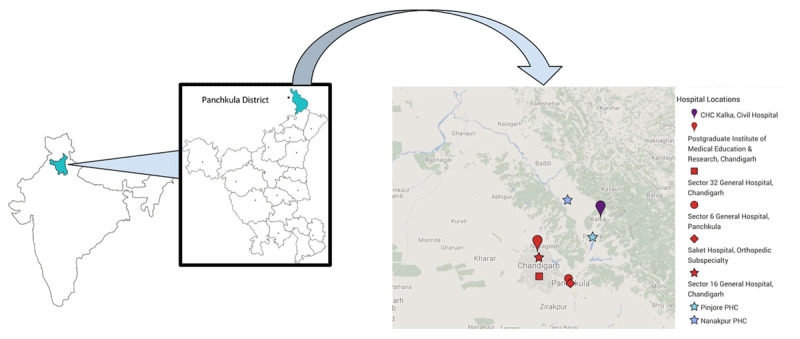
This map of government health facilities was created through Google Maps in 2016. The facilities in red are the tertiary facilities. Patients access the tertiary facilities after referral from the CHC in purple. The distances from the CHC to the tertiary facilities range from 19.31 km to 30.35 km.

Indian healthcare facilities range from private to public, rural to urban, and most importantly, from primary to tertiary. The state manages the public sector workforce with national managerial and financial support. There are 20 hospitals within a 15 km radius of Nanakpur. The radius of 15 km was chosen to capitalize on the golden hour of trauma because even in a car, this distance takes 60 minutes to travel due to the traffic, available routes, and road conditions. Most people of Nanakpur would have to walk, take the bus or hitchhike parts of that distance, which thereby increases the travel time. The rural population of Nanakpur mainly secures healthcare from public health facilities [21]; therefore, only these were included in this study (***Figure 1***).

**Figure 2:**
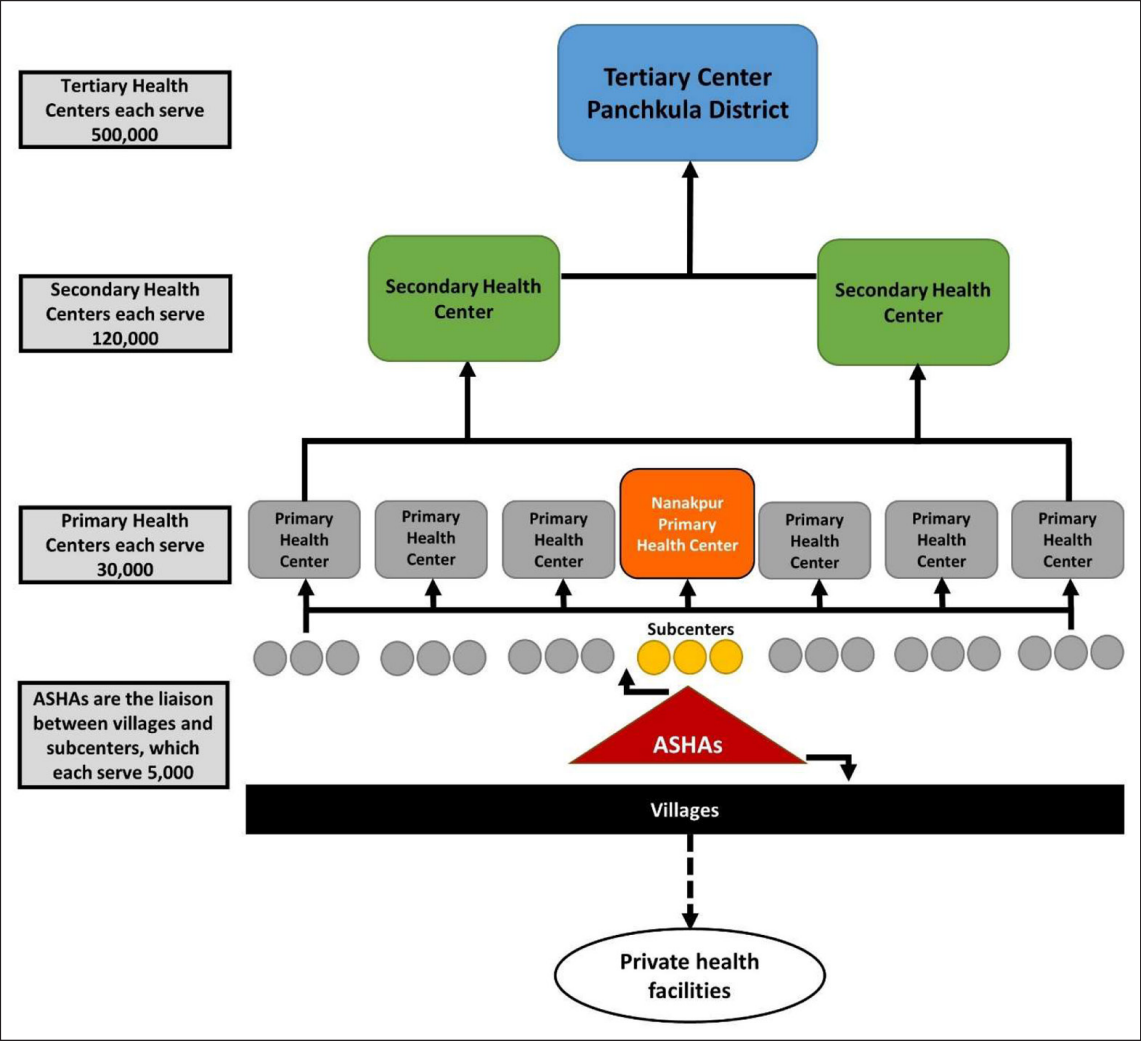
Hierarchy of government healthcare facilities in rural India.

Public health facilities in India are organized according to a hierarchy developed by the NRHM (***Figure 2***). Subcenters (SC) provide the most basic level of care, with each SC serving a population of roughly 5,000 (***Table 1***). According to the NRHM guidelines, the SC is the first point of contact between the primary healthcare system and the community. Primary Health Centers (PHC) serve a population of approximately 30,000 and are required to have six observation beds and at least one physician. The secondary health centers, Community Health Centers (CHC), are the first level referral centers and are considered “District Hospitals,” as defined by the World Health Organization (WHO). Government civil hospitals represent the highest level of care within the public system. These tertiary-care facilities are divided into two categories, with the smaller acting as the first point of care for most urban patients. Tertiary centers are meant to serve 85–95% of the medical needs of their district and on an average day, have at least an 80% bed occupancy rate [18].

**Table 1:** Requirements of each level of government healthcare and the surveyed facilities serving Nanakpur. * Secondary level of care represents World Health Organization district level hospital. ** AYUSH Physician: Ayurveda, Yoga and Naturopathy, Unani, Siddha and Homeopathy physician.


LEVEL OF CARE	HEALTH FACILITY	POPULATION SERVED	MANDATORY WORKFORCE	BEDS	NANAKPUR HEALTH FACILITY

	Subcenter	5,000	1 male healthcare worker	N/A	

			1 female healthcare worker		

Primary	Primary Health Center	30,000	1 General Medicine	6	PHC Nankpur

Secondary*	Community Health Center	120,000	1 General Surgeon	30	PHC Pinjore
	
			1 General Medicine		CHC Kalka

			1 Obstetrician		

			1 Pediatrician		

			1 Anesthesiologist		

Tertiary	Sub-district hospital	500,000	1 General Surgeon	31–100	

			1 General Medicine		

			1 Obstetrician		

			1 Pediatrician		

			1 Anesthesiologist		

			1 Ophthalmologist		

			1 Orthopedic Surgeon		

			1 ENT Surgeon		

			1 Radiologist		

			1 Dental Surgeon		

			7 General Duty Doctors		

			1 Public Health Manager		

			1 AYUSH Physician**		

			1 Hospital Superintendent		

Tertiary	District Hospital	32,000–3,000,000	2–4 General Surgeon	101–500	Sector 6 General Hospital, Panchkula

			2–5 General Medicine		

			2–6 Obstetricians		
	
			2–5 Pediatricians		Sector 16 General Hospital, Chandigarh

			2–4 Anesthesiologists		

			1–2 Ophthalmologist		
	
			1–2 Orthopedic Surgeon		Sector 32 General Hospital, Chandigarh

			1–2 ENT Surgeon		

			1–2 Radiologist		
	
			1–3 Dental Surgeon		Post Graduate Institute of Medical Education and Research, Chandigarh

			1–4 Pathologist		

			11–23 Medical Officers		

			0–1 Dermatologist		

			1 Psychiatrist		

			0–1 Microbiologist		

			0–1 Forensic Specialist		

			1 AYUSH Physician**		


The present study was conducted in coordination with the Nanakpur PHC and the Panchkula Civil Surgeon’s office. Inclusion criteria were collaboratively agreed upon to be government-run health facilities serving the population of Nanakpur with at least one operating room (OR). Private health facilities were excluded. A total of eight facilities were included in this assessment: Nanakpur PHC, Pinjore PHC, Kalka CHC, Sector 6 General Hospital, Sector 16 General Hospital, Sector 32 Government Hospital, Postgraduate Institute of Medical Education and Research, and Saket Orthopedic Hospital.

### Survey Instruments

The PIPES and INTACT survey instruments were utilized to assess surgical and trauma care capacities, respectively. The PIPES survey is a modification of the WHO Tool for Situational Analysis to Assess Emergency and Essential Surgical Care (TSAAEESC). It reduced the total number of data points collected from 256 to 105 and replaced the scaled-scoring system with a binary scoring system [22,23,24,25,26,27]. The INTACT tool was created subsequently using components of PIPES specific to trauma care, totaling 40 items. Use of cervical collars is the only INTACT item not originally present in the PIPES survey [23]. The paper tool lists the category and has an “always available/not always available” response system such that “always available” is scored as “1” and “not always available” is scored as “0.” The binary system allows a total score to be calculated for both PIPES and INTACT tools, easing data comparison from different countries and/or time periods.

A combined PIPES and INTACT survey instrument, originally created for a separate study in Bolivia, was used [28]. This revised tool included modified equipment and supplies sections. All equipment and supply items originally present in TSAAEESC were included in the survey, as well as the added response option of “sometimes available.” For the purposes of the present study, however, only results from the 106 PIPES and INTACT survey items are discussed. To maintain the PIPES and INTACT binary response, “sometimes available” was scored as “not always available” or “0.”

### Data Collection

Data collection occurred over a two-week period in June 2015. Site visits were conducted, and either a local physician, nurse, or hospital administrator completed the paper survey in English. Procedural logs were provided by six of the eight facilities over varying time periods (month to year) to supplement responses to items in the procedures section of the survey. Procedural records from Sector 16 General Hospital and Saket Orthopedic Hospital were not available.

### Data Analysis

The PIPES and INTACT tools are divided into five subsections: personnel, infrastructure, procedures, equipment and supplies. Subsection and overall index scores were calculated for both tools. Standard to the tools, the index score is the sum of the total items divided by the number of items in the survey instrument and then multiplied by 10. Higher PIPES or INTACT indices correspond to greater surgical or trauma care capacity, respectively.

For the present study, the PIPES personnel item “general doctors who do surgery” was interpreted differently at each facility, so it was omitted from the final analysis. The Saket Orthopedic Hospital was excluded from analysis because it only had orthopedic components of the PIPES and INTACT tools.

Data from paper forms were entered into Microsoft Excel (Microsoft Excel Professional 2013, version 15.0, Redmond, WA: Microsoft Corporation). Statistical Analysis Software (SAS Institute Inc., version 9.4, Cary, NC) was used for statistical analysis. Medians and interquartile ranges (IQR) were calculated for individual items, subsection scores, and overall PIPES and INTACT indices. Facilities were then divided into two groups for comparison: tertiary level versus PHC/CHC levels. Medians between groups were compared via Wilcoxon Rank sum tests. A p-value ≤ 0.05 was considered significant.

## Results

Of the seven facilities included in the analysis, three were primary or secondary level centers and four were tertiary level centers. Both PIPES and INTACT index scores and individual subsection scores demonstrated similar trends, with surgical and trauma care capabilities increasing with higher levels of care (***Table 2***). The PIPES index score was significantly higher for tertiary facilities than for PHCs and CHCs [13.8 (IQR 9.5, 18.2) vs. 4.7 (IQR 3.9, 6.2), p = 0.03]. The INTACT index scores demonstrated a similar trend with tertiary facilities scoring significantly higher than PHCs and CHCs [9.5 (IQR 8.3, 9.9) vs. 3.8 (IQR 3.5, 6.3), p = 0.03]. Though the trends in these scores was expected, the main differences among these seven facilities with operating rooms were in the personnel and procedure categories.

**Table 2:** Individual and index median PIPES and INTACT scores divided by health facility size. * The p-values were determined with the Wilcoxon rank-sum test.


PIPES	PHC/CHC (N = 3)	TERTIARY (N = 4)	P-VALUE*	INTACT	PHC/CHC (N = 3)	TERTIARY (N = 4)	P-VALUE*

PIPES Index	4.7 (IQR 3.9, 6.2)	13.8 (IQR 9.5, 18.2)	0.03	INTACT Index (IQR)	3.8 (IQR 3.5, 6.3)	9.5 (IQR 8.3, 9.9)	0.03

Personnel	0 (IQR 0, 1.0)	37.5 (IQR 12.5, 71.5)	0.03	Personnel (IQR)	0 (IQR 0, 1.0)	2.0 (IQR 2.0, 2.0)	0.03

Infrastructure	6.0 (IQR 4.0, 10.0)	25.5 (IQR 16.5, 33)	0.03	Infrastructure (IQR)	2.0 (IQR 2.0, 4.0)	7 (IQR 7.0, 7.0)	0.02

Procedures	5.0 (IQR 5.0, 13.0)	37.0 (IQR 30.0, 38.5)	0.03	Procedures (IQR)	4.0 (IQR 3.0, 7.0)	14.5 (IQR 11.5, 15.5)	0.03

Equipment	14.0 (IQR 13.0, 18.0)	21.5 (IQR 17.0, 22.0)	0.21	Equipment (IQR)	6.0 (IQR 5.0, 9.0)	10.5 (IQR 8.5, 11)	0.07

Supplies	19.0 (IQR 18.0, 22.0)	23.5 (IQR 22.0, 24.5)	0.08	Supplies (IQR)	4.0 (IQR 3.0, 4.0)	4.0 (IQR 4.0, 4.0)	0.25


### Workforce

A total of 79 general surgeons and 90 anesthesiologists work at the seven surveyed facilities, with a majority located at the tertiary level facilities. Neither the PHCs nor the CHC had a general surgeon.

### Infrastructure

All included facilities had water and electricity, as well as medical records and a laboratory to test blood and urine (***Table 3***). Assessed facilities had between six and 1,948 beds, and a median of 3.0 (IQR 0.0, 20.0) operating rooms per facility [29]. The PHCs had non-functional ORs. The tertiary health facilities were the only centers with functional computerized axial tomography scanners, functional ultrasound machines, and a staffed intensive care unit.

**Table 3:** Comparison of median infrastructure values of the PHCs/CHC vs. tertiary health centers. * The p-values were determined with the Wilcoxon rank-sum test.


INFRASTRUCTURE ITEM	PHC/CHCS (N = 3)	TERTIARY HEALTH CENTERS (N = 4)	P-VALUE*

Running/Portable Water	1.0 (IQR 1.0, 1.0)	1.0 (IQR 1.0, 1.0)	0.99

Electricity	1.0 (IQR 1.0, 1.0)	1.0 (IQR 1.0, 1.0)	0.99

Back-up Generator	1.0 (IQR 0, 1.0)	1.0 (IQR 0.5, 1.0)	0.82

Incinerator	0 (IQR 0, 0)	0.5 (IQR 0, 1.0)	0.18

Medical Records	1.0 (IQR 1.0, 1.0)	1.0 (IQR 1.0, 1.0)	0.99

Emergency Room	1.0 (IQR 0, 1.0)	1.0 (IQR 1.0, 1.0)	0.25

Postoperative Care Area	0 (IQR 0, 1.0)	1.0 (IQR 1.0, 1.0)	0.07

Intensive Care Unit	0 (IQR 0, 0)	1.0 (IQR 1.0, 1.0)	0.01

Blood Bank	0 (IQR 0, 1.0)	1.0 (IQR 1.0, 1.0)	0.07

Lab to Test Blood and Urine	1.0 (IQR 1.0, 1.0)	1.0 (IQR 1.0, 1.0)	0.99

Functioning X-Ray Machine	0 (IQR 0, 1.0)	1.0 (IQR 1.0, 1.0)	0.07

Functioning Ultrasound Machine	0 (IQR 0, 0)	1.0 (IQR 1.0, 1.0)	0.01

Functioning CT Scanner	0 (IQR 0, 0)	1.0 (IQR 1.0, 1.0)	0.01

Number of ORs	0 (IQR 0, 1.0)	13.0 (IQR 4.5, 20.5)	0.03


### Procedures

Of the 40 included procedures, only two were performed at all seven facilities: wound debridement and splinting. The tertiary facilities performed a greater number of procedures than the PHCs and CHCs [37, (IQR 30.0, 38) vs. 5.0 (IQR 5, 13); p = 0.03]. Lower-level facilities did not perform pediatric surgeries, including circumcision, cleft lip or palate, clubfoot, abdominal wall defects, or hernia repairs. All four tertiary hospitals and the CHC administered spinal anesthesia. Tertiary hospitals were the only facilities capable of administering general anesthesia.

Additional procedural data obtained from facility records demonstrated a concentration of surgical services at the tertiary facilities (***Table 4***). Lower-level facilities performed a high number of vaginal deliveries, but few surgical procedures. Of the 39 surgical procedures performed at the CHC in May 2015, 35 (90%) were cesarean sections. In contrast, three of the four tertiary facilities completed a median of 43,902 surgeries annually, or approximately 3,659 surgical procedures monthly.

**Table 4:** The total number of major and minor surgical procedures at each facility.


Health Facility	Time period	Total Number of Vaginal Deliveries	Total Number of Surgical Procedures

Nanakpur PHC	May 2015	20	0

Pinjore PHC	June 2015	12	0

Kalka CHC	May 2015	99	39

Sector 6 General Hospital	2013	N/A	7402

Sector 32 General Hospital	2014	N/A	43902

Postgraduate Institute of Medical Education and Research	2013	N/A	182177


### Equipment and Supplies

Most equipment and supplies items were available at all facility levels. Universally available supplies ranged from stethoscopes and thermometers to procedural equipment such as adult and pediatric endotracheal tubes and compressed oxygen. Surgery-specific equipment was available at the CHC and all four tertiary level hospitals, including adult and pediatric oropharyngeal airways, anesthesia machines, pulse oximeters, and surgical instrument sets. However, there were some deficits even at the tertiary facilities, where not all were equipped with a pediatric Macintosh laryngoscope or a cricothyroidotomy set.

## Discussion

This is the first published assessment of surgical and trauma capacities of the rural Nanakpur region in India via the PIPES and INTACT survey instruments. Deficiencies were most evident in personnel and available procedures at the PHC and CHC levels. The scarcity of resources at lower levels of care leads to delays in patients receiving surgical and trauma care, where time is of the essence. Yet there are likely many potential factors contributing to patients not receiving needed care.

According to the interdisciplinary framework applied in the maternal health field, the first delay is in seeking care, the second in reaching care and the third in receiving care [7]. The first delay, a product of financial and geographic restrictions, level of education, and disconnect from formal health systems, was not formally assessed in this study. However, this study did assess aspects of the second and third delays, which were consequences of limited surgical capacity in the region and inadequate resources at a specific facility, respectively. Although the PHC and CHC level facilities were equipped for surgical procedures with basic infrastructure and surgical supplies, no general surgeons were active at the time of this study.

To address the second and third delays in receiving care from a health system standpoint, interventions to improve access to basic surgical and trauma care should target the PHCs, which were not operating at full-capacity as demonstrated by non-functioning ORs and few physicians. Because these facilities individually serve the smallest population, up to 30,000 people, they recognize the specific demographics and needs of their communities, and directly oversee the work of the Accredited Social Health Activists (ASHAs), the Indian community health workers. Community members also have easiest access to these facilities as they are often located within the community. Currently, PHCs refer their complicated deliveries, cesarean sections, and high-level trauma to the CHC, increasing the burden at the over-crowded secondary and tertiary level facilities. However, with minimal investment in personnel and infrastructure maintenance, the PHCs, already outfitted with an OR, could become the site for 80–90% of basic surgical procedures and initial trauma stabilization [7].

The concentration of healthcare personnel in the urban centers directly contrasts with deficiencies in healthcare personnel at the PHC and CHC level. The country increased the number of medical schools from 270 in 2008 to 419 in 2015, in an effort to increase the overall healthcare workforce [21]. India further mandated that all Bachelor of Medicine and Bachelor of Surgery students complete a one year rural rotation to mitigate the scarcity of resources in rural areas. Accordingly, young physicians and medical students could be systematically incentivized with professional gains such as entrance into professional societies to work in rural areas, thereby reducing second and third delays and encouraging physician investment in the community [30].

A more immediate solution could focus on training the ASHAs and nurses as non-physician surgical and anesthesia providers, addressing the first and second delays [31,32]. The ASHAs are trusted local community women who travel door-to-door to provide antenatal education, increase access to immunizations, and promote institutional deliveries. The community health worker model has documented success in Haiti, Bangladesh, and other areas of India [7]. In particular, the NRHM’s ASHA program has improved maternal and child health through regular prenatal and postpartum care, and increased hospital delivery rate [33]. Expanding the role of ASHAs could strengthen the prehospital network with increased manpower [32,34]. A trauma-specific intervention could engage local physicians to teach an existing first responder training course to ASHAs in order to safely stabilize patients at the scene of injury and transport them safely to more definitive care. A formalized training program would leverage ASHAs in stabilizing patients and guiding them to the appropriate level of care [7,35].

### Limitations

There are several limitations to this study. Our data are restricted to a specific period and responses were subjective, based on a single respondent from each facility. The ability to perform certain procedures was not confirmed by observation or records, and timeframes were not consistent among procedure logs. Furthermore, information was not gathered on the quantity of specific equipment or supplies, or specific personnel’s ability to use certain equipment or supply items. The question “number of doctors performing surgery” was not uniformly interpreted as any physician who performed surgery and may have been interpreted as a doctor with surgical training. The confusion led to elimination of the question from analysis because of the high response variability. The omission highlights a potential language and culture barrier. Although all the doctors and staff who completed the survey spoke English, they may not have understood survey questions as intended and may not have felt comfortable asking foreign students for clarification. Another limitation was the small size of the region. Although this quality allowed the PIPES and INTACT tools to be applied at all healthcare levels to gain a better understanding of the government health facility hierarchy, the size limits the ability to extrapolate these results to all areas of India.

### Future Studies

Future assessments should collect the total number of procedures completed at each facility over the same time period to correlate with the snapshot data that the PIPES and INTACT surveys capture. Future studies could also expand the sample area to the state of Haryana or cluster sample the entire country. More health facilities, including private facilities, should be included so operating theatre, surgeon, and surgical procedure densities can be calculated. These can then be compared across different regions of Haryana, states in India, and globally to better understand the current state and future directions of surgical care in India.

## Conclusions

Surgical and trauma care are integral parts of public health systems. This study represents the first use of PIPES and INTACT tools in the region and likely reflects the health care capacities of other rural areas in India. Though not surprising, surgical and trauma capacity weaknesses were most evident in personnel and procedures at the PHC and CHC healthcare facility levels. These results will direct future low-cost initiatives through the lens of the three-delay model. This includes reducing the first delay in seeking care by implementation of first responder courses to empower laypeople in providing basic trauma care. Increasing the number of trained healthcare and non-healthcare surgical personnel, like the ASHAs, at the PHC and CHC level can improve access to safe surgical care for the people of Nanakpur. Ultimately, these initiatives will strengthen the overall healthcare capacity of the region.
